# English- and Spanish-Speaking Patient Preferences on Home Blood Pressure Monitors in an Urban Safety Net Setting: Qualitative Study

**DOI:** 10.2196/60196

**Published:** 2025-08-29

**Authors:** Jonathan J Shih, Vivian E Kwok, Isabel Luna, Hyunjin Cindy Kim, Faviola Garcia, Christian Gutierrez, Mahal Garcia, Courtney R Lyles, Elaine C Khoong

**Affiliations:** 1 School of Medicine, University of California, San Francisco, 513 Parnassus Avenue, San Francisco, CA, 94143, United States; 2 Department of Medicine, Division of General Internal Medicine, San Francisco General Hospital, University of California, San Francisco, 2540 23rd Street, Room 4708, San Francisco, CA, 94110, United States, 1 415-502-6300; 3 UCSF Action Research Center for Health Equity, San Francisco General Hospital, San Francisco, CA, United States; 4 Center for Healthcare Policy and Research, University of California, Davis, Davis, CA, United States; 5 Department of Public Health Science, University of California, Davis, Davis, CA, United States

**Keywords:** telemedicine, telehealth, monitoring, blood pressure monitoring, hypertension, patient preference, healthcare disparities, safety-net providers, physiologic, ambulatory

## Abstract

**Background:**

Self-measured blood pressure monitoring is necessary for successful management of hypertension. However, disparities in blood pressure control persist, with low-income patients and racial and ethnic minorities more likely to have uncontrolled hypertension. These patients are also at increased risk for digital exclusion. Several validated blood pressure monitors for self-measured monitoring are available, but little is known about patient preferences between different device traits. Studies have shown that poor usability or technology design can lead to barriers to adoption.

**Objective:**

We investigated patient-reported barriers, preferences, and facilitators to self-measured blood pressure monitoring from a diverse population at an urban safety-net hospital.

**Methods:**

This qualitative study included English- and Spanish-speaking patients with hypertension. Participants completed a survey about sociodemographic traits, self-measured blood pressure monitoring practices and training, and experience with technology. Semi-structured interviews were conducted to elicit preferences about blood pressure devices, the accompanying mobile apps, and their experience sharing blood pressure measurements with their providers. Interviews included participant demonstration of home blood pressure measurement to evaluate baseline self-measured blood pressure monitoring technique. Two home blood pressure monitoring devices were presented: a Bluetooth-enabled device and a cellular-enabled device that syncs data directly. Surveys and interviews were conducted in participants’ preferred language. Rapid qualitative data analysis was applied to analyze qualitative data.

**Results:**

Fifteen participants (8 English-speaking and 7 Spanish-speaking) were enrolled. Participants all identified as racial and ethnic minorities. Educational attainment varied, ranging from less than high school to college graduates. Eight exhibited some form of digital inaccessibility: lacking internet access, not activating their patient portal, or having difficulty connecting a device to Wi-Fi. Most required assistance with Bluetooth pairing and navigating app features. Overall, participants valued tracking their blood pressure, were motivated to engage in self-measured blood pressure monitoring practices, and desired training. Nearly all participants demonstrated inconsistencies in blood pressure education, displayed incorrect measurement techniques, and had not received formal training on self-measured blood pressure monitoring. Spanish-speaking participants reported that using apps was challenging because they were presented in English and wanted translated apps and resources. The cost of features was a key factor in device preference.

**Conclusions:**

Patient-reported barriers to successful self-measured blood pressure monitoring adoption include cost, insufficient training, digital inaccessibility, and language discordance. Addressing these challenges may enhance the adoption of self-measured blood pressure monitoring in safety net populations. Providers should evaluate patients’ preferences and develop tailored interventions when recommending self-measured blood pressure monitoring. Cellular self-measured blood pressure monitoring devices that automatically transmit blood pressure readings may reduce digital complexity and promote sharing results with providers, though future studies are needed to evaluate usability and implementation.

## Introduction

Hypertension is highly prevalent, resulting in significant cardiovascular disease morbidity and mortality [1-3]. Self-measured blood pressure monitoring (SMBP) is an evidence-based guideline-recommended strategy for improving hypertension management [4-6], in which patients regularly check their own blood pressure (BP) at home and share these results with their clinical care team to improve hypertension control. This approach has shown promising results in populations with worse hypertension outcomes, such as individuals with low income or from racial and ethnic minority backgrounds [7-11].

Despite its potential impact, SMBP faces implementation challenges, especially in populations and health care centers where hypertension disparities are most pronounced. These populations, including Black, Latine/Hispanic, and Asian adults; those with limited insurance; individuals with lower educational attainment; and those with limited English proficiency have worse BP control [8,9,12,13].

Few studies have investigated patient perspectives on challenges to and preferences for SMBP monitoring [14-16], especially in safety net health systems where providers “organize and deliver a significant level of health care and other health-related services to uninsured, Medicaid, and other vulnerable patients” [17]. In addition, these studies do not explore in-detail preferences for home BP monitors and do not assess home measurement technique. Perspectives from patients receiving care in safety net settings are crucial for designing effective SMBP interventions tailored to communities experiencing disparities in BP control. To address the gap in knowledge on patient-reported barriers to SMBP, preferences for different types of BP monitors, and facilitators that would support SMBP in a racially diverse and low-income population, we conducted a qualitative, observational study of patients with hypertension receiving care at an urban safety net hospital.

## Methods

### Study Sample

Participants included English- and Spanish-speaking patients with a diagnosis of hypertension receiving care from an urban academic safety net system. Participants were recruited from August 2022 through October 2023 from an outpatient registry of patients with hypertension. Patients were called and invited to share information about their use of home BP monitors. Participant interviews were not connected to hospital visits. Patients were excluded if primary care clinicians thought the patients would be unable to provide consent due to cognitive impairment or capacity. Participants were recruited until data saturation was reached, as no new themes emerged during the interviews [18]. This study follows the SRQR (Standards for Reporting Qualitative Research) guidelines [19].

### Survey and Interview Administration

All participants completed a survey and a semistructured interview in their preferred language with a native speaker. Researchers followed an interview guide (Multimedia Appendix 1 and Multimedia Appendix 2), which was updated based on iterative review and feedback from the study team and included participant demonstration of home BP measurement to evaluate baseline SMBP technique. The survey included closed-ended questions about sociodemographic traits, SMBP practices and training, and experience with technology. Participants self-reported demographic information, including gender, race and ethnicity, highest educational attainment, and preferred language. A survey question was added mid-study to further assess participants’ digital literacy. Interviews asked open-ended questions about BP device preferences, experience with BP device mobile apps, and current practices around tracking and sharing BP measurements with their clinicians. Each participant was presented with 2 home BP monitoring devices during the interview: a Bluetooth-enabled device (Device 1) and a cellular-enabled device that syncs data directly (Device 2). Participants were allotted 10 minutes per device to test and record a BP reading. Interviews were audio-recorded, and interviewers simultaneously documented field notes to capture participants’ nonverbal cues, key observations, and immediate reflections on the interview content.

### Analysis

Rapid qualitative data analysis was used to analyze qualitative data [20-23]. Compared to traditional qualitative methods, this is a rigorous, pragmatic approach to analyzing qualitative data on an accelerated timeline. The data collection and analysis goals of rapid qualitative analysis focus on identifying key descriptors to address time-sensitive questions as opposed to developing a theoretically extensive understanding of a concept. As this study sought to inform intervention elements for future home BP monitoring trials [24-26], rapid qualitative analysis with purposeful data reduction activities was used. Rapid qualitative data analysis steps include developing a codebook of domains from interviews, summarizing interviews based on domains, and validating across the study team for consistency.

Analysis involved an iterative and systematic approach to identifying key insights from the interview data. An interview guide (MultimediaAppendices 1  2) was initially developed to gather feedback relevant to intervention design, which was iteratively updated to ensure subsequent interviews captured all relevant perspectives and content areas. To standardize interview analyses, a summary template was structured around 8 initial coding domains that were developed according to these emerging topics: (1) Existing general knowledge of and prior training about blood pressure, (2) Impact of device’s physical features on patient preference, (3) Considerations and technique when measuring their blood pressure at home, (4) Patient preferences: Bluetooth versus cellular connection, (5) Logging and sharing BP data, (6) User app experience: Viewing BP results and navigating features, (7) Evaluation of provided training and learning materials, and (8) Ideal training and resource preferences.

Following this initial coding and summarization process, we engaged in a second phase of analysis focused on data reduction and theme generation. Through iterative team discussions, we collapsed the 8 domains into 3 higher-order codes, each representing a major conceptual area of patient experience with SMBP. Specifically, domains 1, 7, and 8 were merged into Code 1: Patient Knowledge about SMBP. Domains 3, 5, and 6 were consolidated into Code 2: Patterns and Challenges in Self-Monitoring Blood Pressure. Domains 2 and 4 formed Code 3: Varied Patient Preferences on Home Blood Pressure Monitoring Device Features. Within each analytic code, we then identified themes, defined as recurrent, explanatory patterns that captured participants’ reported barriers, preferences, and facilitators for SMBP adoption. This multistep process allowed us to move systematically from coded content to broader thematic insights. After this process, we selected representative quotes from the interview transcripts to illustrate key concepts within each code.

Three investigators (JS, VK, and IL) reviewed audio recordings and field notes independently for an initial subset of interviews. Using the template with the set of 8 domains, each interview was summarized to extract key concepts. The process continued for 8 interviews until consensus was reached on the approach and content of the interview summaries. The remaining interviews were then analyzed and summarized by a single investigator using the established framework (IL). After completing summaries from all interviews, the content within each domain was reviewed across all interviews to identify overarching themes. Particular attention was given to differences in responses by language to capture any nuanced variations. Agreement on key themes was reached through multiple collaborative discussions with the entire research team until consensus was achieved. Frequency of participant responses within each theme for these nonsurvey interview data was synthesized according to the following nomenclature: “few” representing approximately 3-5 participants, “many” representing 5-7, “most” representing the majority or at least 8 of the 15 total participants, and “nearly all” representing 13 or 14.

Analytical credibility and trustworthiness were enhanced through multiple strategies [27]. We used investigator triangulation with 3 independent coders (JS, VK, and IL) analyzing initial interviews and coordinated with clinical staff members who had years of experience onboarding patients to SMBP studies. Two senior study investigators (CL and EK) have conducted SMBP research for more than 5 years, providing prolonged engagement with the research topic. Interviews lasted more than 90 minutes and incorporated multiple follow-up questions to ensure depth of data collection and participant discussion. Emerging domains were incorporated into the codebook through iterative discussion and team consensus, allowing the analysis to evolve and reflect new insights gained throughout the process.

To ensure dependability and confirmability, we maintained comprehensive audit trails throughout the research process. This included detailed field notes from each interview, multiple versions of our codebook documenting its evolution, and iterations of the interview guide as it was refined based on emerging insights. Meeting minutes from team discussions captured analytical decisions and rationale for theme development. To enhance confirmability, multiple team members independently reviewed the coding process to ensure findings were grounded in participant data rather than researcher assumptions. At least 2 researchers reviewed each transcript, with discrepancies resolved by a third investigator or the whole research team. This audit trail and multireviewer approach helped ensure consistency of findings over time and minimized individual bias in interpretation [27]. Reflexivity was maintained through field notes documenting the researcher’s observations, reactions, and preliminary interpretations during each interview. Research team meetings included structured self-reflection on how our professional backgrounds as healthcare researchers and clinicians in safety-net settings might influence data interpretation. Team members explicitly discussed potential biases related to our experiences with health disparities and digital health implementation. These discussions were used to ensure interpretations remained grounded in participant data.

### Ethical Considerations

All participants provided written informed consent and received a US $50 gift card for participation. Data were deidentified for analysis and reporting. Participants were given the option to withdraw at any time throughout the study and have their data removed. This study was approved by the University of California, San Francisco Institutional Review Board (number 21‐33711).

## Results

### Participant Demographics and Baseline Experience With Technology and Self-Measured Blood Pressure

Table 1 displays the characteristics of 15 total participants, all of whom completed both the survey and interview. Eleven participants were assessed for their SMBP technique against a rubric using the updated interview guide. The median age of participants was 57 (IQR 37-71) years.

From survey responses, all participants had a smartphone, 10 used mobile apps several times a day, and 10 reported no difficulty using their smartphone or installing apps without assistance. Eight participants either did not have internet service at home other than via smartphone, did not know how to connect a device to Wi-Fi, or had not activated their patient portal account.

**Table 1.** Participant demographics and experience with technology.

Characteristics	Values, n (%; N=15)
Female	9 (60)
Race and Ethnicity	
American Indian or Native American	1 (7)
Asian or Pacific Islander	1 (7)
Black or African American	4 (27)
Hispanic or Latine	8 (53)
Two or more	1 (7)
Highest educational attainment	
Less than high school	5 (33)
High school graduate or GED^a^	5 (33)
College graduate or more	5 (33)
Preferred language	
English	8 (53)
Spanish	7 (47)
Experience with technology	
Frequency of using apps for any purpose on phone	
Several times a day	10 (67)
At least once a day	3 (20)
Once a week	2 (13)
Difficulty installing apps on phone	
Not difficult	10 (67)
Somewhat/Very difficult	5 (33)
Has internet service at home other than via smartphone	13 (87)
Activated patient portal account	8 (53)
Difficulty using phone without someone else’s help	
Not difficult	12 (80)
Somewhat/Very difficult	3 (20)
Knows how to connect device to Wi-Fi, (N=11)^b^	6 (55)

a GED: General Educational Development.

b This question was later added to the survey to further assess digital literacy

In the past 12 months, 5 participants measured their BP outside of the clinic, all at home using their own BP monitor. Overall, 3 measured their BP less than once a month, 2 measured at least once a month, and 2 shared these BP measurements with their clinical team. Four participants reported that measuring BP at home and sharing results with their clinician was “extremely helpful.”

Through our analysis, we consolidated the 8 initial coding domains into 3 high-order analytical codes. Within each analytical code, we identified themes that captured the key barriers, preferences, and facilitators for SMBP adoption. All analytical codes, themes, and notable quotes are provided in Table 2.

**Table 2.** Analytical codes, themes, and notable quotes from interviews.

Themes	Description	Notable quotes
Analytical Code 1: Patient Knowledge about SMBP^a^		
Theme 1.1 Inconsistencies and Gaps Exist in Patient Education	Few participants received adequate education on home BP^b^ monitoring techniques	“Drawings and pictures help people understand faster…People never really go through books [of instructions], something simpler would be better.” “[There is] a lot of writing… [I would] need to read more than once.” “It’s impossible to get accurate reading at home.” “It would’ve taken me a bit more time [without a demonstration].”
Theme 1.2: Patients Desire Training and Education	All participants desired more in-person training and education, and in particular take-home materials that were easy to understand and language-concordant	
Theme 1.3: Incorrect Technique and Missing Considerations in Home BP Measurement	Few participants engaged in all recommended practices for accurate home measurement	
Analytical Code 2: Patterns and Challenges in Self-Monitoring Blood Pressure		
Theme 2.1 Participants Like the Idea of Tracking BP	While most participants did not track their BP, they thought it was important and liked the apps’ ability to do so	“Yes it is [important to keep a log of your BP readings]. You can talk to your doctor about it and things going on in your life.” "You would keep track of your blood pressure, you know that day it was a little high, now it’s better. You would keep better track of your medication.” “Really the main thing I liked [about the apps] is … how you can go on the app and you can get to your blood pressure feed. Just that fast... [You don’t have to] go through a whole bunch of stuff. It’s just right there. That’s what I like about it.” “[Logging in the app is] really good because every time I write it down, I always forget where I put it. I’m always losing it. It’s great that my [BP recordings] stay [in the app]. I can just show it. I’d love that.” “You meet your doctor once every 3 months, so if I take a reading now, I forget it after two weeks.”
Theme 2.2: Participants Experience Difficulty With Using the Apps	Many participants had difficulty using the apps, including patients who reported no prior difficulty with smartphone app usage. Translating apps into Spanish would enhance usability for Spanish-speaking participants	
Theme 2.3: Participants Do Not Share their BP With Providers	Most participants did not currently share their BP. Some tracked BP, but did not share with their provider due to misplaced logs.	
Analytical Code 3: Varied Patient Preferences on Home Blood Pressure Monitoring Device Features		
Theme 3.1: Device Features Did Not Impact Overall Device Preference	Participants differed on preferences for BP cuff type and device size, but these features did not impact device preference.	“I don’t want anything gigantic. I want it to be perfectly small, where I can take it if I need be, to be able to take it with me if I’m traveling.” “This is pretty big. So it won’t be good for traveling too much.” “Just sending a message – if it’s going to cost you money – that’s a rip off.” “[Paying for remote patient monitoring] is a turn off. If I had to pay more money, I’d rather not.” “If it can be sent to my [doctor] without me knowing, that’d be great… [But I don’t want to be] paying for [that].” “The only thing holding me back from [this device] is paying.” “People don’t have access to batteries, like you or the manufacturer think. You think an old man of 70‐80 [years of age] would go out to buy a battery?”
Theme 3.2: Participants Require Assistance With Bluetooth Pairing	Many participants needed help pairing the Bluetooth device, and some preferred the non-Bluetooth option because of this requirement	
Theme 3.3: Cost as a Deciding Factor in Device Preference	While participants preferred BP results to be automatically shared with their provider, they would not pay for this feature and also had concerns related to the cost of batteries.	

a SMBP: Self-measured blood pressure monitoring

b BP: blood pressure.

### Analytical Code 1: Patient Knowledge About SMBP

#### Inconsistencies and Gaps Exist in Patient Education

Many reported no formal training on using their BP monitor or normal BP range. Instead, many learned by observing providers in clinics, reading the manual, or watching online video tutorials. A few participants received demonstrations or were told about BP at clinic visits but did not remember what they learned and were not given additional materials.

Existing knowledge about BP was incomplete and varied. Few participants reported understanding the idea of normal BP range. Some participants noted the importance of taking repeated measurements, staying calm before a reading, and the importance of weight and diet for BP management.

#### Patients Desire Training and Education

Nearly all participants wanted more training about BP devices and at-home measurement, preferring in-person demonstrations. Some said written instructions or video tutorials may suffice. In addition to training on BP device usage, some participants wanted information about BP ranges. Nearly all participants expected clinic support if they encountered an issue when measuring BP. Some participants also mentioned troubleshooting with family.

Participants valued having additional written and video resources. They preferred written resources that were easy to read and understand, larger with large and bolded font, and contained concise, numbered steps to follow. Some participants also thought visual illustrations would be helpful. Participants noted that the instruction manuals included with the BP monitoring devices were detailed and had a lot of information, which some felt was overwhelming. Spanish-speaking participants wanted to have Spanish materials.

#### Incorrect Technique and Missing Considerations in Home BP Measurement

**Figure 1.**
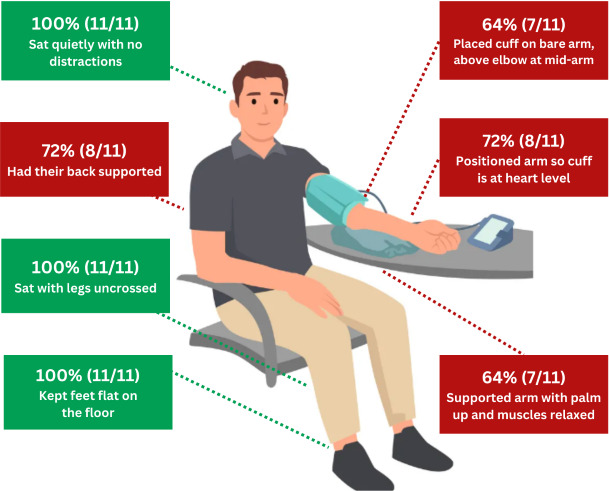
Demonstration of blood pressure measurement technique evaluated by American Medical Association and American Heart Association guidelines.

Most participants did not follow all guidelines set forth by the American Heart Association (AHA) and American Medical Association (AMA) for accurately measuring BP at home [28] and displayed incorrect technique when demonstrating an at-home BP measurement (Figure 1). Of the 11 participants who were asked to demonstrate how they would measure their BP at home, only 4 were consistent with all guidelines set forth by the AMA and AHA. The most common missed techniques in BP measurement were placing the cuff on the bare arm, above the elbow at midarm; ensuring the arm is supported, with palm up and muscles relaxed; positioning the arm at heart level; and ensuring the back is supported. All participants properly uncrossed their legs, rested their feet flatly on the floor, and sat quietly without distractions (though we provided the environment).

When asked about considerations when measuring BP at home, nearly all participants did not consider the time of day or the timing of medications, eating meals, smoking, drinking alcohol, or using the restroom in relation to their readings. Most participants, however, did report resting or relaxing prior to taking a measurement.

### Analytical Code 2: Patterns and Challenges in Self-Monitoring Blood Pressure

#### Participants Like the Idea of Tracking BP

Most participants did not currently track their BP but cited it as important or wanted to do so. Some would track BP only under particular circumstances, such as when their BP is unusually high.

Participants liked that the apps linked and tracked their BP results from the device. Nearly all participants appreciated visual aids (eg, graphs) that show their BP history. Many participants liked immediately seeing their results when opening the app; some participants specified that Device 2’s app was simpler and straightforward to use. Some also appreciated additional features of Device 1’s app, such as the option of keeping a diary to write notes. Participants appreciated visuals, a straightforward interface that enables easy app usage and BP tracking, and personalized tracking capabilities.

#### Participants Experienced Difficulty With Using the Apps

Difficulty in app usage varied. When surveyed, all participants who reported difficulty using a smartphone without assistance or installing apps also had difficulty navigating app features or required assistance upon observation. Some who reported no difficulty in these 2 survey questions also had difficulty or required assistance upon demonstration. Nearly all participants who had trouble navigating the apps were Spanish-speaking and expressed that translating apps entirely into Spanish would enhance ease of use.

#### Participants Do Not Share Their BP With Providers

Furthermore, most participants did not currently share their BP with their provider. Most participants who track their BP often misplace their measurement logs. Two participants shared with their providers only if they remembered where they kept their readings.

### Analytical Code 3: Varied Patient Preferences on Home Blood Pressure Monitoring Device Features

#### Device Features Did Not Impact Overall Device Preference

Participants prioritize comfort and ease of use when evaluating 2 different BP cuffs, but this preference was split between hard and soft cuffs. Preferences also differed between the smaller size of Device 2 and the larger display of Device 1. However, cuff type and portability of the devices did not impact overall device preference.

#### Participants Require Assistance With Bluetooth Pairing

Many participants did not view Bluetooth pairing, a feature of Device 1, as a deterrent to measuring BP, with half of them citing familiarity with Bluetooth. We also observed that these participants successfully connected the device with Bluetooth. Nearly all other participants required help from study staff to pair the Bluetooth device. A few explicitly cited not wanting to deal with pairing or repairing Bluetooth. Some preferred Device 2 because it did not require Bluetooth or said that Device 1 was more difficult to use for this reason.

#### Cost as a Deciding Factor in Device Preference

Moreover, while approximately half of the participants value having their BP results automatically shared with their provider (such as would occur in a cellular-enabled monitor like Device 2), nearly all would not pay for this feature. One was open to having results automatically sent if in poor health. A few would pay US $5-US $15 a month if required, but they were strongly opposed. If this feature were free, three participants would prefer Device 2. Most strongly preferred having a plug-in charging option for the battery-operated devices because of concerns related to accessing or purchasing future batteries.

## Discussion

### Principal Findings

In our qualitative study of safety net patients with hypertension, the cost of device features, gaps in existing BP knowledge, and lack of training and resources presented challenges to SMBP adoption. Our findings highlight the need to provide affordable, language-concordant resources and comprehensive training to leverage SMBP for hypertension management in safety net patients.

Our findings of participant motivation for SMBP and barriers related to cost and health literacy among low-income and minority communities were overall consistent with prior studies [11,14-16]. Our participants valued tracking their BP and are motivated to engage in SMBP practices and share results with their providers [11]. Moreover, participants preferred having their readings automatically shared with their provider without requiring pairing to their own device, citing it as extremely helpful. However, nearly all participants were not willing to pay for this feature. Plug-in devices were also strongly preferred to avoid the cost of batteries. These findings suggest that SMBP adoption in lower-income and uninsured and Medicaid populations is impacted by affordability or payor coverage of SMBP devices that meet patients’ needs. In addition, nearly all patients demonstrated inconsistencies in BP education, displayed incorrect BP measurement technique, and had not received formal training. These findings reinforce the notion that barriers to successful SMBP adoption stem from external care factors, such as cost and gaps in available training and resources, rather than patient motivation. Providers should evaluate patients’ barriers and preferences when recommending SMBP.

In addition to cost, digital accessibility and literacy should be assessed as contributors to SMBP non-adoption. Aligned with studies that demonstrate socioeconomic status and Medicaid insurance as risk factors for digital exclusion [29], 8 of 15 participants exhibited some form of digital inaccessibility: lacking internet access, not activating their patient portal, or having difficulty connecting a device to Wi-Fi. Furthermore, most participants required assistance with pairing Bluetooth to Device 1 and with navigating app features on both devices, potentially indicating limited digital literacy. SMBP interventions should be complemented with patient training and resources. Importantly, it appears that cellular SMBP devices that automatically transmit BP readings to reduce digital complexity may promote sharing results with providers, and this should be considered as a focus of future research and implementation.

Moreover, Spanish-speaking participants reported that using apps was challenging because they were presented in English. This further supports the unmet need to have user-friendly, language-concordant digital SMBP tools [11,30]. Spanish-speaking participants also wanted manuals and training to be delivered in Spanish. Addressing language nonconcordance in training and resources for other prominent but less prevalent languages (eg, Arabic) [31] in addition to Spanish may further promote widespread SMBP adoption. Notably, patients who face barriers in digital literacy and language discordance may be especially vulnerable to SMBP adoption challenges.

While a previous study on home BP monitoring enrolled both English and Spanish speakers [15], our study was the first to assess patient preferences between 2 SMBP devices and the impact of language on SMBP practices. Our results further add to knowledge about patient preferences for communication modality of their BP results with their care team and barriers to SMBP among racially diverse, low-income populations.

### Limitations

Our study was limited by a small, convenient sample. However, our sample size aligns with findings from a recent systematic review suggesting that saturation in qualitative research can often be achieved within a narrow sample size of 9-17 interviews [18]. Participants were assessed at only 1 time point; SMBP skills and preferences may differ in real care scenarios. In addition, despite our efforts to ensure credibility through independent coding, team discussions, and participant validation of findings, researcher interpretation may still reflect inherent subject biases common to qualitative research. While we maintained reflexivity through field notes and team discussions about our positionality, as with all qualitative work, the lived experiences and perspectives of the study team, which included individuals with expertise in primary care, health equity, and public health, may have introduced implicit biases in theme identification. Although we described our study setting and participants in detail to support transferability, our single-site study within an urban safety net context may limit applicability to different health care settings. Future studies should explore how support systems (eg, family and caregivers) could impact SMBP adoption. Collaborating directly with patients to refine analytical domains and themes could also improve the transferability of our findings. An ongoing randomized controlled trial is currently underway to longitudinally assess device implementation and BP outcomes, which will help address these limitations [25].

### Conclusions

Patients’ values and barriers can inform solutions that facilitate and improve patient self-management of hypertension. Our findings reinforce the importance of affordability, accessibility, and providing robust resources when implementing SMBP in diverse, safety net populations. The effectiveness of cellular-enabled SMBP devices should be further evaluated.

## Supplementary material

10.2196/60196 Multimedia Appendix 1 Interview guide in English.

10.2196/60196 Multimedia Appendix 2 Interview guide in Spanish.

## Abbreviations

AHA: American Heart Association
AMA: American Medical Association
BP: blood pressure
SMBP: self-measured blood pressure monitoring
