# Supplementary material for: English- and Spanish-Speaking Patient Preferences on Home Blood Pressure Monitors in an Urban Safety Net Setting: Qualitative Study
Source: JMIR Cardio. 2025 Aug 29;9:e60196. doi: 10.2196/60196 (PMC12396792; doi:10.2196/60196)
Supplement: Multimedia Appendix 1 [file cardio-v9-e60196-s001.docx]

**Interview Guide: Patient Preferences on Blood Pressure Cuff Usage**

Note: We will only be recruiting patients with a confirmed diagnosis of hypertension.

Key:

*Italics =* what interviewer reads

**Bold** = Instructions to the interviewer

Probes = written as probe

# Survey questions should be done remotely. If completed, in-person questions begin here. Please ask if subject has any questions and answer appropriately. If not, remind that we will be recording and begin the audio recording on Zoom with closed captioning and backup audio recording on phone. No video recording of subject allowed.

# Baseline Training

*“To start, can you share what training you’ve had about using a blood pressure device or about blood pressure ranges?“*

1. *“What were you taught?”*
   1. Probe: Did they include any materials (i.e. videos, infographics)? What were you shown?
   2. Probe: What did you learn?
2. *“How were you trained?*
   1. Probe: Was it in-person in the doctor’s office, did they show you or just give you handouts or was it online?
   2. Probe: Who did the training?
3. *“About how often do you get trained?”*
4. *“How long was the training?*
5. *“What did you like about your training? What could have made it better?”*
   1. Probe: Did you feel confident leaving that training?
   2. Probe: Would you want any follow-up training? If so, what would that look like?

# Demonstrating devices and assessing taking BP at home

”*Now, we’re going to move onto these blood pressure devices. Let’s first show you the two devices and cuffs.”*

1. **Show both devices and cuffs to the patient**
   1. *“What are your very first impressions of these blood pressure* ***devices****”?*
      1. Probe: *“What do you think about the weight?*
         1. Probe: “*What do you think about the portability, such as taking it on travel?*
      2. Probe: “*What do you think about the Omron device being able to be plugged in or use batteries versus the Care Simple device, which can only use batteries*
      3. Probe: “*What do you think about the different* ***cuffs*** *in particular?”*
2. *“Before we have you try the devices, we want to ask: What are things you do or avoid to make sure you’re getting an accurate blood pressure reading at home?”*
   1. Probe: *“Do you take it at the same time each day?*
   2. Probe: *“Does when you eat or what you eat change when you take your blood pressure? What about any medications? Or drinking or smoking?”*
   3. Probe: *“Do you rest before taking a reading? If so, how long?”*
   4. Probe: *“Do you use the restroom/bathroom before taking your blood pressure?*
3. *“We also wanted to discuss how these blood pressure devices’ mobile app can show and track your blood pressure readings. With the app, patients can have a record of their blood pressure to show or send to their health care providers.”*
   1. *“Is this something you would want? Being able to connect your results to an app on the phone to see your results? etc.*

**NOTE: If participant number = odd, start with Omron. If participant number = even, start with CareSimple. Note on field guide which was first device.**

*“We’ll now show you how to set up this device on your phone and how to access the app. In the clinic, a nurse will typically show and set this up for you as well, so I’ll do that now.*

**OMRON**

1. *“This device connects to your phone via Bluetooth. The device also comes with written instructions.* **Flip to page XX to show them instructions.**
2. *“As mentioned, this device will show and track your blood pressure readings on this app on your phone. This is how you would connect it to your phone.”* **Begin Bluetooth connection.**
   1. **Once connected, walk through the following items in the Omron app:**
      1. Dashboard (this is where patients can see their last BP reading. Be sure to distinguish between systolic/diastolic BP, pulse, and the meaning behind #s; show them My Diary, which lets them track symptoms and what they ate)
      2. History (this is where patients can see a list of ALL BP readings; show how they can view these as a list or as a chart)
      3. Settings (show how they can pair the app to phone)
      4. How to enter readings manually
   2. **Make sure to reset/unpair Omron device so patients can demonstrate taking BP from the start.**
   3. **Go to Step 6.**

**CARESIMPLE**

1. *“This is a cellular blood pressure device, and a nurse will typically have this device set up for you. We would enroll you, and you would receive an email or text to download the app and enroll with your username.* ***You will not need to connect it to your phone like you would with a device that uses Bluetooth.*** *As we mentioned, this device will show and track your blood pressure readings on this app. Results are directly sent to a phone number rather than through Bluetooth.”*
   1. **Walk through the following items in the CareSimple app:**
      1. Home (this is where patients can see their last BP reading. Be sure to distinguish between systolic/diastolic BP and pulse)
      2. History (to view history, **go to Profile 🡪 Settings 🡪 Logbook**)
      3. Inbox (this is where patients would get reminders to take BP)
      4. How to enter readings manually
   2. **Go to Step 6.**

**For each device, give patients 10 minutes at most for steps 6 and 7 (this includes the time the device takes for the BP reading). Only provide assistance if explicitly asked or if 5 minutes have passed and patient has not yet started taking the reading.**

1. *“Now we’d like to have you try this device. Could you show me how you would take your blood pressure at home and find the results in the app? I know this could be an unfamiliar device, but try your best for now. This is not a test at all.”*
   1. **If starting with Omron, ensure that the patient also completes Bluetooth pairing.**
   2. **Refer to Field Notes and check-off actions that the patient completes when measuring BP. Note any difficulties that the patient encounters regarding pairing or taking BP in the Field Notes.**
2. **Prompt if necessary:** “*Now,* *can you try pairing/opening/syncing the app and viewing your results?”*
   1. **Have the patient also demonstrate back viewing the Dashboard/Home, History, Settings, Inbox, and entering readings manually for each device.**
   2. **Note in Field Notes if patient has trouble accessing app.**
3. **Ask these questions after patient has obtained reading and accessed app.**
   1. *“Was there anything challenging about using this device or app?*
   2. *“Was there anything you liked about using the device or app?*
   3. *“How did you feel about the training or materials that were provided in the package?”*
      1. *Probe: “What did you like about them?”*
      2. *Probe: “What do you think is missing or could be improved?”*

**Repeat above steps 6-8 with the other device. The BP checklist does not need to be completed again, but note any discrepancies in how patient takes their BP compared to the other device.**

1. *“Now we will go over the second device’s app and see you use this second device to take your BP.”*

# Comparing devices and assessing preferences

1. ***Hand patient the summary device summary sheet.*** *“Now that you’ve used both devices, we wanted to review again some of the differences between them and what you are able to do with each of them:*
   1. *The* ***Omron*** *device is a Bluetooth device, which means it will connect to your phone so that your results will show on the app.*
   2. *The* ***Care Simple*** *device is a cellular blood pressure device, which does not need to connect to your phone to show your results on the app. Since the Care Simple device is a cellular one, it requires an additional cell phone plan.*
   3. *Both devices will show you a history of your blood pressure readings in the app.*
      1. *For the* ***Omron*** *device, you can tell your health care provider your blood pressure readings when you go in for appointments, or if you communicate with them for example through MyChart.*
      2. *The* ***Care Simple*** *device will automatically send your results to a system that your provider can access.“*
2. *“Did you have any preference for one device over the other? Tell me more about that.”*
   1. **Ask following additional questions if patient does not already cover these themes:**
      1. Physical design:
         1. **Encourage patient to hold device.** *“Did you consider weight or portability in your preferences? Is that something important to you when choosing a device?*
         2. *How about the cuff design and comfort?*
         3. *How about whether the device uses batteries only versus can be plugged in?”*
      2. App/pairing:
         1. *How did you find the Bluetooth pairing to be?*
            1. *Do you think having to occasionally re-pair the Bluetooth would discourage you from taking or logging your BP?*
            2. Probe: *What about if you had re-pair it every time you take your blood pressure, for say– weeks? Months? Years?*
         2. *Do you find it important to keep a log of your blood pressure readings?*
            1. *Do you currently log your blood pressure?*
         3. *How did you feel about the app?*
            1. *Did you find it important to be able to track the data in the app?*
            2. *Was it easy or hard to use?*

Probe If English is not their language preference: Would it be useful to have it in your preferred language?

- - - - 1. *What was missing that you would’ve liked to see?*
        2. *What information did you think was excessive?*
    1. Sharing with provider
       1. *Do you think it’s important that you share your blood pressure readings with your provider?*
          1. *(If not already covered) Probe: Do you do this now (if not already covered)?*

*Probe: How do you share data now?*

- - - 1. *When do you prefer to share these with providers?*
         1. Probe: *Only in person, or all the time?*
      2. *Would you prefer to have these sent automatically to a system that your provider can access, or would you like to choose when you send your blood pressure readings?*
         1. Probe: *The cellular CareSimple device automatically sends your results to a system that your provider can access. The Omron does not. Does that affect your preferences?*
    1. Paying for a cellular line
       1. *“Would you be willing to pay for a cell phone line (or an additional line to my existing plan) for your results to be automatically sent to a system that your provider can access?*
       2. *How much would you be willing to pay/how long?*

1. *What kind of training or materials—like any manual, instructions, or pamphlets that come with the device--would you want for the Omron device?*
   1. *Would you be able to use it at home by yourself?*
      1. Probe if English is not their language preference: *Would it be helpful to have training materials provided to you in a different language? Why or why not?*
   2. *What kind of training would you want for the CareSimple device?*
      1. *Would you be able to use it at home by yourself?*
      2. Probe if English is not their language preference: *Would it be helpful to have training materials provided to you in a different language? Why or why not?*

# Training/Manual (After BOTH devices)

1. *“Would you like to receive training or materials as part of your onboarding for a new blood pressure device? If not, why not?*
2. [If yes] “*What would your ideal training be like?*
   1. Probe: “*How often would you like to be trained?*
   2. Probe: “*Would you prefer virtual or in person training?*
   3. Probe: “*Do you think it would be nice to have additional training after your first initial one?*
   4. Probe: “*What format for training would be most helpful? Messages, videos, in-person from doctor or nurse, or pamphlets*
3. *“Do you expect the clinic to provide you with training?*
4. *“What training would you expect when you run into a problem?*
   1. Probe: “*Would you be able to figure out the problem with, for example, written instructions, a video tutorial, or help from family or friends?*
   2. Probe: “*Would you expect the clinic to help?”*

# Conclude patient piloting interview

*“Those are all the questions we had. Thank you for taking the time to talk through your experience with the blood pressure devices. We are hopeful that this will help patients, especially to save time by cutting down on in-person appointments. Do you have any comments or questions?”*
