# Supplementary material for: English- and Spanish-Speaking Patient Preferences on Home Blood Pressure Monitors in an Urban Safety Net Setting: Qualitative Study
Source: JMIR Cardio. 2025 Aug 29;9:e60196. doi: 10.2196/60196 (PMC12396792; doi:10.2196/60196)
Supplement: Multimedia Appendix 2 [file cardio-v9-e60196-s002.docx]

**(SPANISH) Interview Guide: Patient Preferences on Blood Pressure Cuff Usage**

Note: We will only be recruiting patients with a confirmed diagnosis of hypertension.

Key:

*Italics =* what interviewer reads

**Bold** = Instructions to the interviewer

Probes = written as probe

**Survey questions should be done remotely. If completed, in-person questions begin here. Please ask if subject has any questions and answer appropriately. If not, remind that we will be recording and begin the audio recording on Zoom with closed captioning and backup audio recording on phone. No video recording of subject allowed.**

**Baseline Training**

*"Para empezar, ¿puede compartir qué entrenamiento ha recibido sobre el uso de un dispositivo de presión arterial o sobre los rangos de presión arterial?"*

1. "¿Qué te enseñaron?"
2. Probe: ¿Le mostraron algún material (por ejemplo, vídeos, infografías)? ¿Qué le mostraron?
3. Probe: ¿Qué ha aprendido?
4. "¿Cómo te entrenaron?
5. Probe: ¿Fue en persona en una consulta médica, te lo enseñaron o simplemente te dieron folletos, o fue por Internet?
6. Probe: ¿Quién te ha entrenado?
7. *”¿Con qué frecuencia recibe entrenamiento?"*
8. *“¿Cuánto duró el entrenamiento?”*
9. *"¿Qué le ha gustado de su entrenamiento? ¿Qué podría haberlo hecho mejor?"*
10. Probe: ¿Se sintió seguro al final de el entrenamiento?
11. Probe: ¿Desea algún tipo de entrenamiento de seguimiento? En caso afirmativo, ¿cómo sería?

**Demonstrating devices and assessing taking BP at home**

​​*“Ahora, vamos a seguir con estos aparatos de presión arterial. Primero vamos a enseñarle los dos aparatos y mangas.”*

1. **Show both devices and cuffs to the patient**
2. *“*¿*Cuáles son sus primeras impresiones sobre estos tensiómetros??*
3. Probe: *"¿Qué opinas del peso?*
4. Probe: *"¿Qué opina de la portabilidad, como para llevárselo de viaje?”*
5. Probe: “*¿Qué opina de que el dispositivo Omron se pueda conectar o utilizar pilas frente al dispositivo Care Simple, que sólo puede utilizar pilas?”*
6. Probe: “*¿Qué opinas de los diferentes manguitos en particular?”*

1. *“Antes de que pruebe los dispositivos, queremos preguntarle: ¿Qué cosas hace o evita para asegurarse de que obtiene una medición precisa de la presión arterial en casa?".*
2. Probe: *“¿Lo tomas a la misma hora todos los días?”*
3. Probe: *"¿Cambia el momento en que come o lo que come cuando se toma la presión arterial? ¿Y los medicamentos? ¿O al beber o fumar?”*
4. Probe: *“¿Descansa antes de hacer una medición? Si es así, ¿cuánto tiempo?"*
5. Probe: *"¿Usa el baño antes de medirse la presión arterial?”*

1. *"También queríamos hablar de cómo la aplicación móvil de estos dispositivos de presión arterial puede mostrar y realizar un seguimiento de sus mediciones de presión arterial. Con la aplicación, los pacientes pueden tener un registro de su presión arterial para mostrar o enviar a sus proveedores de atención médica."*
2. *"¿Es esto algo que te gustaría? Poder conectar tus resultados a una aplicación en el teléfono para ver tus resultados?*

**NOTE: If participant number = odd, start with Omron. If participant number = even, start with CareSimple. Note on field guide which was first device.** 
 
*"Ahora le mostraremos cómo configurar este dispositivo en su teléfono y cómo acceder a la aplicación. En la clínica, una enfermera suele mostrar y configurar esto para usted también, así que voy a hacer eso ahora.*

**OMRON** 

1. *"Este dispositivo se conecta a su teléfono a través de Bluetooth. El dispositivo también viene con instrucciones escritas.* **Flip to page XX to show them instructions.**
2. *"Como se ha mencionado, este dispositivo mostrará y hará un seguimiento de tus mediciones de presión arterial en esta aplicación en tu teléfono. Así es como lo conectarías a tu teléfono.”* **Begin Bluetooth connection.**
3. **Once connected, walk through the following items in the Omron app:**
4. Panel de aplicación (aquí es donde los pacientes pueden ver su última lectura de presión arterial. Asegúrese de distinguir entre presión arterial sistólica/diastólica, pulso y el significado de los números; muéstreles Mi diario, que les permite hacer un seguimiento de los síntomas y de lo que han comido).
5. Historia (aquí es donde los pacientes pueden ver una lista de TODAS las mediciones de presión arterial; muéstreles cómo pueden verlas en forma de lista o de gráfico).
6. Configuración (muestra cómo emparejar la aplicación con el teléfono)
7. Cómo introducir lecturas manualmente
8. **Make sure to reset/unpair Omron device so patients can demonstrate taking BP from the start.**
9. **Go to Step 6.**

**CARESIMPLE**

1. *"Se trata de un dispositivo celular para medir la tensión arterial, que normalmente le configura una enfermera. Le inscribiremos y recibirá un correo electrónico o un mensaje de texto para que descargue la aplicación y se inscriba con su nombre de usuario.* ***Usted no tendrá que conectarlo a su teléfono como lo haría con un dispositivo que utiliza Bluetooth****. Como hemos mencionado, este dispositivo mostrará y realizará un seguimiento de sus lecturas de presión arterial en esta aplicación. Los resultados se envían directamente a un número de teléfono en lugar de a través de Bluetooth".*
2. **Walk through the following items in the CareSimple app:**
3. Inicio (aquí es donde los pacientes pueden ver su última medición de la presión arterial. Asegúrese de distinguir entre presión sistólica/diastólica y pulso).
4. Historial (para ver su historial de mediciones, **go to Profile à Settings à Logbook**)
5. Bandeja de entrada (aquí es donde los pacientes recibirían recordatorios para tomar la presión arterial)
6. Cómo introducir mediciones manualmente
7. **Go to Step 6.**

**For each device, give patients 10 minutes at most for steps 6 and 7 (this includes the time the device takes for the BP reading). Only provide assistance if explicitly asked or if 5 minutes have passed and patient has not yet started taking the reading.** 

1. *"Ahora nos gustaría que probara este dispositivo. ¿Podría mostrarme cómo se tomaría la tensión en casa y encontrar los resultados en la aplicación? Sé que puede ser un dispositivo poco familiar, pero inténtalo lo mejor que puedas por ahora. Esto no es una prueba en absoluto".”*
2. **If starting with Omron, ensure that the patient also completes Bluetooth pairing.**
3. **Refer to Field Notes and check-off actions that the patient completes when measuring BP. Note any difficulties that the patient encounters regarding pairing or taking BP in the Field Notes.**

1. **Prompt if necessary:** *"Ahora, ¿puedes intentar emparejar/abrir/sincronizar la aplicación y ver los resultados?”*
2. **Have the patient also demonstrate back viewing the Dashboard/Home, History, Settings, Inbox, and entering readings manually for each device.**
3. **Note in Field Notes if patient has trouble accessing app.**

1. **Ask these questions after patient has obtained reading and accessed app.**
2. *“¿Hubo algún reto al utilizar este dispositivo o aplicación?”*
3. *“¿Hubo algo que le gustó del uso del dispositivo o la aplicación?”*
4. *“¿Qué le pareció la información o el material que se le proporcionó en el paquete?".*
5. *Probe: "¿Qué te ha gustado?"*
6. *Probe: "¿Qué cree que falta o podría mejorarse?"*

**Repeat above steps 6-8 with the other device. The BP checklist does not need to be completed again, but note any discrepancies in how patient takes their BP compared to the other device.**

1. "Ahora vamos a repasar la aplicación del segundo dispositivo y a ver cómo usas este segundo dispositivo para tomarte la presión".

**Comparing devices and assessing preferences**

1. ***Hand patient the summary device summary sheet.*** *"Ahora que ya has utilizado los dos dispositivos, queríamos repasar de nuevo algunas de las diferencias entre ellos y lo que puedes hacer con cada uno de ellos:*
2. *El dispositivo* ***Omron*** *es un dispositivo Bluetooth, lo que significa que se conectará a su teléfono para que sus resultados se muestren en la aplicación.*
3. El dispositivo **Care Simple** es un dispositivo celular de presión arterial, que no necesita conectarse a su teléfono para mostrar sus resultados en la aplicación. Dado que el dispositivo Care Simple es celular, requiere un plan de telefonía móvil adicional.
4. Ambos dispositivos le mostrarán un historial de sus lecturas de presión arterial en la aplicación.
5. En el caso del dispositivo **Omron**, puede informar a su proveedor de atención sanitaria de sus lecturas de tensión arterial cuando acuda a sus citas o si se comunica con él, por ejemplo, a través de MyChart.
6. El dispositivo **Care Simple** enviará automáticamente sus resultados a un sistema al que podrá acceder su proveedor."
7. *"¿Tenía alguna preferencia por un dispositivo u otro? Cuénteme más sobre eso".*
8. **Ask following additional questions if patient does not already cover these themes:**
9. Physical design:
10. **Encourage patient to hold device. Sujete el dispositivo si lo desea.** *¿Tuvo en cuenta el peso o la portabilidad en sus preferencias? ¿Es algo importante para ti a la hora de elegir un dispositivo?*
11. *¿Y el diseño y la comodidad del manguito?*
12. *¿Qué le parece si el aparato funciona sólo con pilas o si se puede conectar a la electricidad?"*
13. App/pairing:
14. *¿Qué te ha parecido el emparejamiento Bluetooth?*
15. *¿Cree que tener que volver a emparejar de vez en cuando el Bluetooth le desanimaría a medir o registrar su presión arterial?*
16. Probe: *¿Y si tuvieras que volver a emparejarlo cada vez que te tomas la presión arterial, durante digamos... semanas? ¿Meses? ¿Años?*
17. *¿Le parece importante llevar un registro de las mediciones de su presión arterial?*
18. *¿Actualmente registra su presión arterial?*
19. *¿Qué le ha parecido la aplicación?*
20. *¿Le pareció importante poder hacer un seguimiento de los datos en la aplicación?*
21. *¿Fue fácil o difícil de usar?*
22. **Probe If English is not their language preference:** ¿Sería útil tenerlo en su idioma preferido?
23. *¿Qué echó en falta que le hubiera gustado ver?*
24. *¿Qué información le pareció excesiva?*

1. Sharing with provider
2. *¿Crees que es importante que compartas tus mediciones de presión arterial con tu proveedor?*
3. *(If not already covered) Probe: ¿Haces esto ahora? (if not already covered)?*
4. *Probe: ¿Cómo se comparten ahora los datos?*
5. ¿Cuándo prefiere compartirlas con los proveedores?
6. Probe: *¿Sólo en persona o todo el tiempo?*
7. *¿Prefiere que se envíen automáticamente a un sistema al que pueda acceder su proveedor, o quiere elegir cuándo envía sus lecturas de tensión arterial?*
8. Probe: *El dispositivo* ***CareSimple*** *celular envía automáticamente sus resultados a un sistema al que puede acceder su proveedor. El Omron no lo hace. ¿Afecta esto a sus preferencias?*
9. Paying for a cellular line
10. *¿Estaría dispuesto a pagar una línea de teléfono móvil (o una línea adicional a mi plan actual) para que sus resultados se envíen automáticamente a un sistema al que pueda acceder su proveedor?*
11. *¿Cuánto estaría dispuesto a pagar/cuánto tiempo?*
12. *¿Qué tipo de formación o materiales -como manuales, instrucciones o folletos- quieres para el dispositivo Omron?*
13. *¿Serías capaz de utilizarlo tú solo en casa??*

b. **Probe if English is not their language preference:** *¿Le sería útil disponer de material de formación en otro idioma? ¿Por qué sí o por qué no?What kind of training would you want for the CareSimple device?*

**Training/Manual (After BOTH devices)**

1. *"¿Le gustaría recibir formación o materiales como parte de su introducción a un nuevo dispositivo de presión arterial? En caso negativo, ¿por qué no?”*
2. ***[En caso afirmativo]*** *"¿Cómo sería tu entrenamiento ideal?*
3. Probe: *"¿Con qué frecuencia le gustaría recibir entrenamiento?”*
4. Probe: “*¿Prefiere el entrenamiento virtual o en persona?”*
5. Probe: *"¿Cree que estaría bien recibir un entrenamiento adicional después del primero inicial?*
6. Probe: “*¿Qué formato de entrenamiento sería más útil? Mensajes, vídeos, en persona por el médico o la enfermera, o folletos?”*
7. *“¿Espera que la clínica proporcione entrenamiento?”*
8. *"¿Qué formación esperarías cuando te encuentras con un problema?*
9. Probe: *"¿Serías capaz de resolver el problema con, por ejemplo, instrucciones escritas, un tutorial en vídeo o la ayuda de familiares o amigos?”*
10. Probe: "¿Esperas que la clínica te ayude?"

**Conclude patient piloting interview**

*"Ésas son todas las preguntas que teníamos. Gracias por dedicar su tiempo a hablarnos de su experiencia con los tensiómetros. Esperamos que esto ayude a los pacientes, especialmente a ahorrar tiempo al reducir las citas en persona. ¿Tiene algún comentario o pregunta?"*
